# Hybrid Metabolic Activity-Related Prognostic Model and Its Effect on Tumor in Renal Cell Carcinoma

**DOI:** 10.1155/2022/1147545

**Published:** 2022-12-21

**Authors:** Lei Yu, Lei Ding, Zhong-Yuan Wang, Xing-Zhi Zhao, Yu-Hao Wang, Chao Liang, Jie Li

**Affiliations:** ^1^Department of Urology, The Affiliated Suqian First People's Hospital of Nanjing Medical University, Suqian, China; ^2^Department of Urology, The First Affiliated Hospital of Nanjing Medical University, Nanjing, China

## Abstract

**Background:**

Tumor cells with a hybrid metabolic state, in which glycolysis and oxidative phosphorylation (OXPHOS) can be used, usually have a strong ability to adapt to different stress environments due to their metabolic plasticity. However, few studies on tumor cells with this phenotype have been conducted in the field of renal cell carcinoma (RCC).

**Methods:**

The metabolic pathway (glycolysis, OXPHOS) related gene sets were obtained from the Molecular Signatures Database (V7.5.1). The gene expression matrix, clinical information, and mutation data were obtained by Perl programming language (5.32.0) mining, the Cancer Genome Atlas and International Cancer Genome Consortium database. Gene Set Enrichment Analysis (GSEA) software (4.0.3) was utilised to analyse glycolysis-related gene sets. Analysis of survival, immune infiltration, mutation, etc. was performed using the R programming language (4.1.0).

**Results:**

Eight genes that are highly associated with glycolysis and OXHPOS were used to construct the cox proportional hazards model, and risk scores were calculated based on this to predict the prognosis of clear cell RCC patients and to classify patients into risk groups. Gene Ontology, the Kyoto Encyclopaedia of Genes and Genomes, and GSEA were analysed according to the differential genes to investigate the signal pathways related to the hybrid metabolic state. Immunoinfiltration analysis revealed that CD8+T cells, M2 macrophages, etc., had significant differences in infiltration. In addition, the analysis of mutation data showed significant differences in the number of mutations of PBRM1, SETD2, and BAP1 between groups. Cell experiments demonstrated that the DLD gene expression was abnormally high in various tumor cells and is associated with the strong migration ability of RCC.

**Conclusions:**

We successfully constructed a risk score system based on glycolysis and OXPHOS-related genes to predict the prognosis of RCC patients. Bioinformatics analysis and cell experiments also revealed the effect of the hybrid metabolic activity on the migration ability and immune activity of RCC and the possible therapeutic targets for patients.

## 1. Introduction

Kidney cancer is the second most common malignancy of the urinary system, ranking 6th in men and 9th in women [1]. The most common pathological types of kidney cancer are clear cell renal cell carcinoma (ccRCC), chromophobe cell RCC, and papillary cell RCC, among which ccRCC accounts for approximately 70% of kidney cancers [2]. Owing to early screening and relatively diversified treatment options, the mortality rate of renal cancer is not high, but some patients are still not sensitive to traditional targeted therapy and immunotherapy [3]. Therefore, the development of potential combination drugs has been a focus in the field of RCC.

Cell metabolism has always been a popular field in malignant tumors, in which glycolysis and oxidative phosphorylation are important metabolic pathways of cells, and their mutual transformation is an important research object in the study of the metabolic plasticity of tumor cells [4]. Studies related to breast cancer have shown that tumor cells with metabolic plasticity have strong metabolic adaptability, tumor proliferation, migration, and invasion ability [5]. Meanwhile, a single-cell sequencing analysis study about RCC suggested that a subcluster exists in tumor cells that have simultaneous glycolysis and oxidative phosphorylation (OXPHOS) activities [6]. In addition, previous studies related to RCC have shown that the transformation of the tumor metabolic status has significant impacts on the tumor immune microenvironment and the efficacy of targeted therapy and immunotherapy [7, 8]. Therefore, the influence of the hybrid metabolic status of tumor cells in RCC on the overall tumor and prognosis of patients should be further investigated.

**Figure 1:**
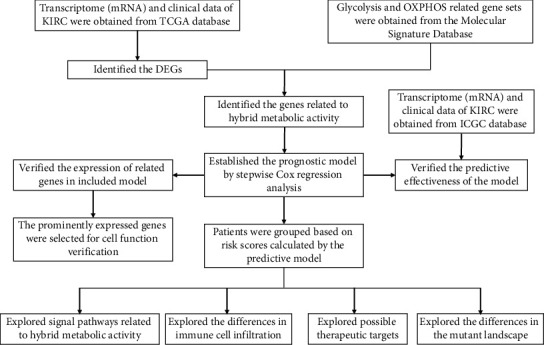
The flow diagram of the present study. TCGA, the Cancer Genome Atlas; DEGs, differentially expressed genes; OXHPOS, oxidative phosphorylation.

Here, we use Pearson correlation coefficient analysis to screen the genes that are highly related to the OXPHOS signal pathway in the glycolysis-related gene set and screen the genes highly related to the glycolysis-signal pathway in the OXPHOS-related gene set in the same way and finally combine the overlapping genes to construct the prognosis model in ccRCC. According to the prognostic model score, patients were divided into high and low-risk groups to further explore the influence of the hybrid metabolic status on RCC. In addition, we further explored the effect of the gene in the prognostic model on the tumor cell function through fundamental experiments (Figure 1). This study may provide new therapeutic targets for ccRCC with hybrid metabolic characteristics.

## 2. Methods

### 2.1. Transcriptional Data and Clinical Information of Patients with ccRCC

The mRNA expression matrix and the clinical information of patients were extracted from the Cancer Genome Atlas (TCGA) database using the Perl programming language (5.32.0) and then standardized and combined by R programming language (4.1.0) [9, 10]. Clinical information was collected including age, sex, grade, stage, TMN stage, duration of survival, and status. The data in this article are available in open databases. We also used the Perl programming language (5.32.0) to collect information on renal cancer from the International Cancer Genome Consortium (ICGC) database with clinical feature data and gene expression matrix. We referred to the methods in the previous literature to standardize and remove the batch effect of these datasets. The results suggest that the gene expression of these data sets has good consistency after merging (Figure S1).

### 2.2. Gene Acquisition of Glycolysis and OXPHOS and Establishment of the Prognostic Model

Gene sets related to glycolysis and OXPHOS are available in the Molecular Signatures Database (MSigDB) (V7.5.1) [11]. We also used the R software package Limma (version 3.40.6) for differential analysis (|logFC| > 1, *P* < 0.05) to obtain the differential genes between tumor tissues and normal tissues. Pearson correlation coefficient analysis was performed on the gene sets of these two metabolic pathways [12]. *P* < 0.05 and |*r*| ≥ 0.3 are the criteria for significant correlation. Finally, stepwise Cox regression using the R software package survival was applied to further screen the genes and construct the prognostic model. The prediction of risk degree is based on the following formula: risk score = ∑_*i*=0_^*n*^Coef(*i*) × *x*(*i*), where Coef (*i*) and *x* (*i*) represent the estimated regression coefficient and the expression of genes, respectively [13].

### 2.3. Evaluation and Validation of the Prognostic Model

Univariate and multivariate cox regression analyses were used to analyse the relationship between the screened genes and prognosis. The Kaplan–Meier (K–M) survival curve was used to visualise the differences in survival between high-risk and low-risk groups. The receiver operating characteristic (ROC) curve was used to assess the accuracy of the prognostic model. Heatmap and scatter plot were used to exhibit the relationship between the risk score and the gene expression and between the risk score and the clinical characteristics, respectively. The nomogram based on multifactor regression analyses integrating risk scores and clinical characteristics was used to determine the extent to which risk scores and clinical features contributed to the outcome variables [14].

### 2.4. Deconvolution of Immune Cell Infiltration in Tumor Microenvironment (TME)

Here, on the basis of our gene expression matrix, the CIBERSORT method was selected to calculate the immunoinfiltrating cell score of each sample using R software package IOBR, a computing tool for immunotumor biology research [15, 16].

### 2.5. Calculation of Mutation Landscape and Microsatellite Instability (MSI) of Tumor

We extracted the mutation data of ccRCC from the TCGA database using the Perl language. A total of 336 samples were tested for mutations, of which 283 (84.2%) were mapped. In addition, we extracted the mutation score, the number of mutations per million bases, for each sample using the Perl programming language. We calculated the MSI score of the ccRCC in the TCGA database using the PreMSIm package in R software [17].

### 2.6. Gene Set Enrichment Analysis

We obtained the Gene Set Enrichment Analysis (GSEA) software (version 3.0) from the GSEA website. We also divided the samples into two groups according to the risk score and downloaded the c2.cp.biocarta.v7.4.symbols.gmt subset from the MsigDB to evaluate the relevant pathways and molecular mechanisms [11, 18]. Meanwhile, we used the R software package Limma to obtain the differential genes (|logFC| > 1, *P* < 0.05) in the high- and low-risk groups and then conducted the Gene Ontology (GO) and Kyoto Encyclopaedia of Genes and Genomes (KEGG) analysis to find the related signal pathways. *P* < 0.05 and FDR < 0.25 were considered statistically significant.

### 2.7. Cell Culture

The in-situ RCC line (786-O), the metastatic RCC lines (Caki-1, Caki-2), and the normal renal tissue cell line (HK-2) were purchased from the Chinese Academy of Sciences (Shanghai, China) and cultured in RMI1640, McCoy's 5A, and F12K (Gibco, USA) medium containing 10% foetal bovine serum, respectively. They were placed in a humidifying incubator containing 5% CO_2_ at 37°C.

### 2.8. Cell Transfection

The RCC cells were transfected with nonspecific miRNA control and DLD siRNA (Hanbio, Shanghai, China) by using Lipofectamine 3000 (Invitrogen, USA) according to the manufacturer's protocol. The transfection sequence used in the experiment was si-DLD (5′- CUUACGCAGAUC AGCCGAU-3′).

### 2.9. Quantitative Real-Time Polymerase Chain Reaction (qRT-PCR)

Using Trizol (Invitrogen, USA) for RNA extraction from the RCC cell lines according to the manufacturer's instructions. miRNA was reverse-transcribed into cDNA using MiR-XTM miRNA first-strand synthesis (Takara, JPN). The total RNA was reversed transcribed into cDNA using PrimeScript RT Master Mix (Takara, JPN). A standard SYBR Green PCR kit (Takara, JPN) was used to perform qRT-PCR. The following forward and reverse primer sequences are shown in Table S1.

### 2.10. Western Blotting

The total proteins of the RCC cells were lysed in RIPA buffer (KeyGene biotech) supplemented with protease inhibitors. Protein was separated by 10% SDS/PAGE after boiling the samples for 15 min. Then, the lysates were transfected onto PVDF membranes in a transfer buffer. The PVDF membranes were blocked in 5% nonfat milk with Tris-buffered saline with Tween (TBST) for 3 h, then the PVDF membranes were treated overnight at 4°C with the following primary antibodies: DLD and GAPDH (Abcam, UK). After cleaning with TBST, the PVDF membranes were treated with the secondary antibody (Abcam, UK) of the corresponding species and finally exposed to the ECL luminometer and collected the image.

### 2.11. Cell Counting Kit-8 (CCK-8) Assay

The cells were placed into the 96-well plate (1.5*∗*10^3^ per well), and 10 *μ*l CCK-8 solution (Beyotime Biotechnology, Shanghai, China) was added into each well at 24, 48, 72, and 96 h. The cells were then incubated at 37°C for 1.5 h and then the optical density was measured at 450 nm by an absorbance reader (Thermo Scientific, USA).

### 2.12. Clone Formation Assay

The 786-O and Caki-1 cells were inoculated into 6-well plates (1.0*∗*10^3^ per well) and incubated at 37°C for 10 days. Afterwards, the cells were washed with 2 ml PBS (Beyotime Biotechnology, Shanghai, China) and fixed with 4% paraformaldehyde (Beyotime Biotechnology, Shanghai, China) and stained by hematoxylin. Each well was observed and photographed by a high-definition camera.

### 2.13. Cell Migration Assay

The Transwell upper chambers were added with 200 *μ*l serum-free medium and 1*∗*10^5^ cells, and then they were placed in a 24-well plate containing 600 ml medium containing 20% fetal bovine serum in each well and cultured at 37°C for 24 h (786-O) and 36 h (Caki-1). The Transwell upper chambers were then rinsed with PBS to remove unmigrated cells and fixed with 4% paraformaldehyde before staining with hematoxylin. Finally, they were observed and photographed under the optical microscope.

### 2.14. Statistical Analysis

We normalized the mRNA expression matrix from the TCGA database through log2 transformation for further analysis. The criterion for the statistically significant difference of all *t*-tests was *P* < 0.05. Statistical analysis and figure drawing were carried out through R software and GraphPad Prism 8.

## 3. Results

### 3.1. Screening Genes Related to Hybrid Metabolic Activity in the Gene Sets of Glycolysis and OXPHOS in ccRCC

**Figure 2:**
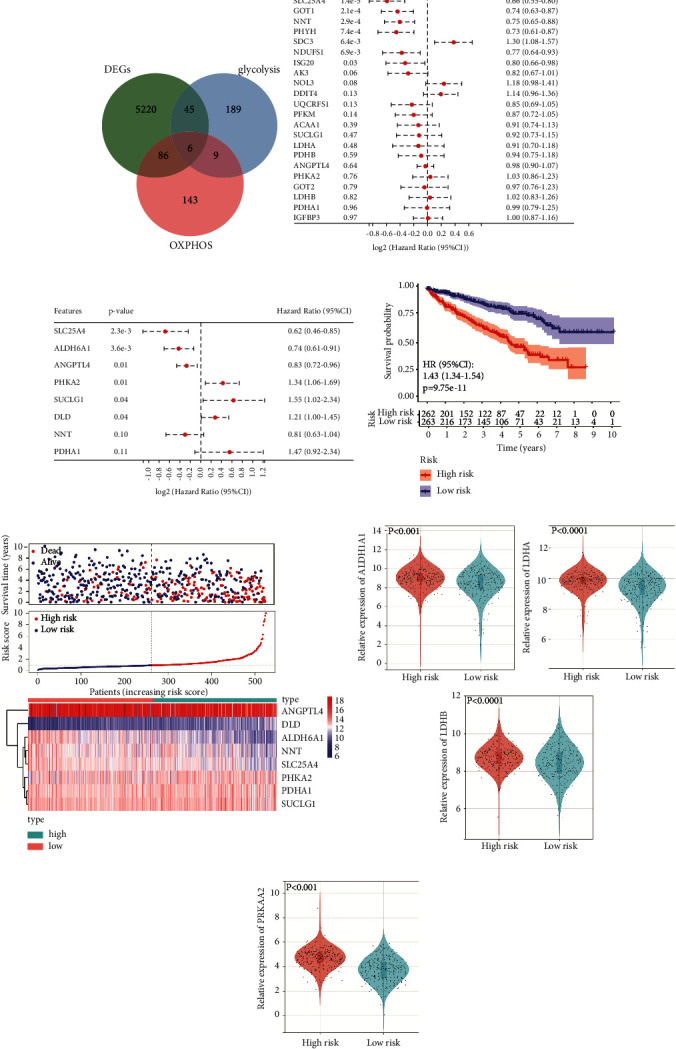
Construction of the hybrid metabolic activity-related prognostic model in the training set. (a) The Venn diagram shows the intersection of glycolysis and oxidative phosphorylation-related gene sets and differential genes in renal cell carcinoma. B&C: the forest map exhibits the results of univariate regression analysis of 26 selected genes associated with hybrid metabolic activity (b) and the multivariate regression model based on these genes screened by stepwise COX regression analysis (c). (d) The K–M curve of patients in high risk and low risk groups. (e) The scatter plot plots the patient's survival time (top) and the distribution of risk scores estimated by the prognostic model (middle). The heatmap (bottom) shows the expression levels of 8 genes involved in the construction of prognosis model in patients in high risk and low risk groups. (f) Violin diagrams of the relationship between the expression of key genes in glycolysis (ALDH1A1, LDHA, and LDHB) (g) and OXPHOS (PRKAA2) (F) metabolic pathways and risk scores.

A total of 525 patients with renal cell carcinoma from the TCGA database were enrolled in the TCGA in the subsequent study, and their corresponding clinical characteristics were shown in Table 1. Based on the gene expression matrix of all the patients with renal cell carcinoma from the TCGA database, we obtained the list of differential genes between cancer and adjacent tissues. We also downloaded gene sets related to glycolysis and OXPHOS from MsigDB and intersected them with the differential gene set (Figure 2(a)). Six duplicate genes were enrolled in the subsequent model construction considering that they were involved in both metabolic pathways. Meanwhile, we analysed the correlation between the differential genes associated with the two metabolic pathways in renal cancer. The correlation between the target genes and the metabolic activity was assessed using the number of genes in the metabolic pathway-related differential gene set that were highly correlated with the target gene (*P* < 0.01 and |*r*| ≥ 0.3). The top 10 genes in the glycolysis-related differential gene set with the highest correlation with OXPHOS and the top 10 genes in the OXPHOS-related differential gene set with the highest correlation with glycolysis were selected. A follow-up analysis was performed based on the above 26 genes.

### 3.2. Establishment and Evaluation of the Prognostic Model Related to Hybrid Metabolic Activity in ccRCC

We first performed univariate regression analysis to observe the relationship between these genes and the survival prognosis of patients with renal cancer and identified nine protective genes and two risk genes (Figure 2(b)). Then, stepwise COX regression analysis was used to further screen the genes and construct the prognostic model related to the hybrid metabolic activity (Figure 2(c)). Finally, a total of eight genes were included in the prediction model, and the coefficients associated with each variable are presented in Table S2. According to the risk score calculated by the prognostic model, the patients were divided into the high- and the low-risk groups and the K–M curves of the two groups illustrate that the survival prognosis of the low-risk group was significantly better than that of the high-risk group (Figure 2(d)). The scatter plot visualises the risk scores of all the patients and exhibits the survival status of the corresponding high- and low-risk groups. The gene expression profiles used to construct the prognostic model were also mapped (Figure 2(e)). In addition, we compared the relationship between the key regulatory genes in the glycolysis pathway (Figure 2(f)) and the OXPHOS pathway (Figure 2(g)) and the risk score and found that the activity of the two metabolic pathways in the high-risk group is higher than that in the low-risk group, suggesting that the tumor cells in these patients were more likely to have a confounding metabolic activity.

**Figure 3:**
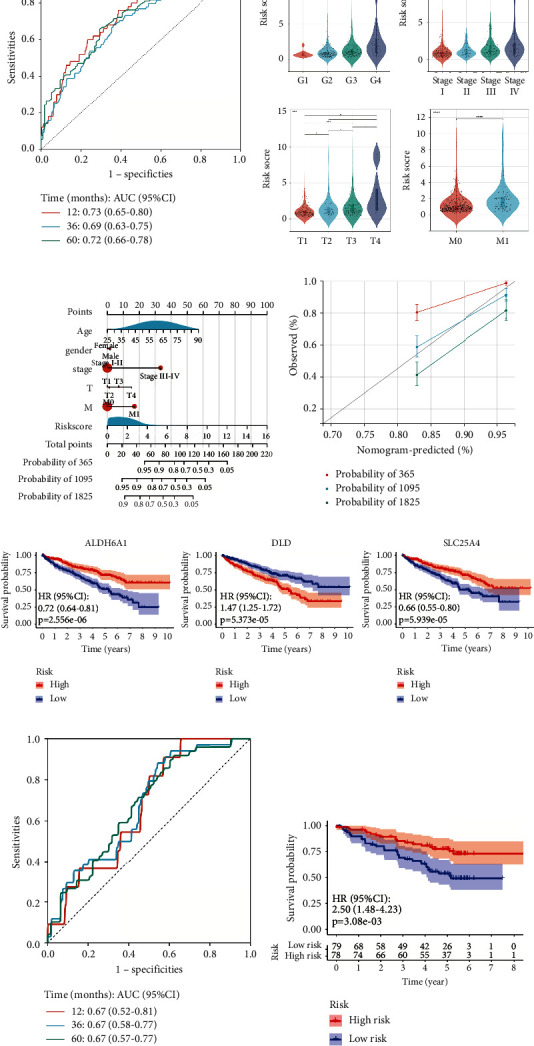
The validation of efficacy of the hybrid metabolic activity-related prognostic model. (a) ROC curves of 1, 3, and 5 years for the hybrid metabolic activity-related prognostic model in the training set. (b) Violin diagrams of the relationship between clinical characteristics (pathological grade, clinical stage, T stage, and tumor metastasis) and risk scores. (c) The nomogram based on clinicopathological parameters and the risk score shows that the risk score accurately predicts 1-, 3- and 5-year survival in patients with RCC (^*∗*^*P* < 0.05, ^*∗∗*^*P* < 0.01, ^*∗∗∗*^*P* < 0.001, ^*∗∗∗∗*^*P* < 0.0001). (d) The calibration curve exhibits the consistency between the observed and predicted overall survival of patients in 1, 3, and 5 years. (e) Patients were grouped according to the expression of ALDH6A1, DLD, SLC25A4, and the corresponding K–M curves were drawn. (f) ROC curves of 1, 3, and 5 years for the hybrid metabolic activity-related prognostic model in the validation set. (g) The K–M curve of patients in high risk and low risk groups in the validation set.

To further evaluate the efficacy of the model, ROC curves were plotted to evaluate the reliability of the risk score in predicting patient survival outcomes in 1-, 3- and 5-year, with all AUCs approaching or exceeding 0.70 (Figure 3(a)). We also grouped the patients according to the pathological grade, clinical stage, and other clinical characteristics of their tumor to compare the risk scores among the groups and observed significant positive associations between them (Figure 3(b)). Finally, we combined the risk score calculated by the hybrid metabolic activity-related prognostic model and the clinical characteristics, including age, gender, clinical stage, T stage, and M stage to construct the nomogram to accurately estimate the 1-, 3-, and 5-year survival and prognosis of these RCC patients. We found that the risk score made an excellent contribution to the prediction of the survival status (Figure 3(c)), and the calibration curve also elucidated that the predicted and real 3-year OS are highly consistent (Figure 3(d)). We collected the survival information and gene expression matrix of 157 patients from the ICGC database and calculated the risk score of each patient with the above model (Table S3). The ROC curve shows that the prediction model still has a certain prediction efficiency in the validation set, and univariate regression analysis suggests that the risk score is a significant risk factor (Figures 3(f) and 3(g)). These results indicate that the prognostic model related to the hybrid metabolic activity has good predictive efficacy.

### 3.3. Investigation of the Signaling Pathways Associated with Hybrid Metabolic Activity in ccRCC

**Figure 4:**
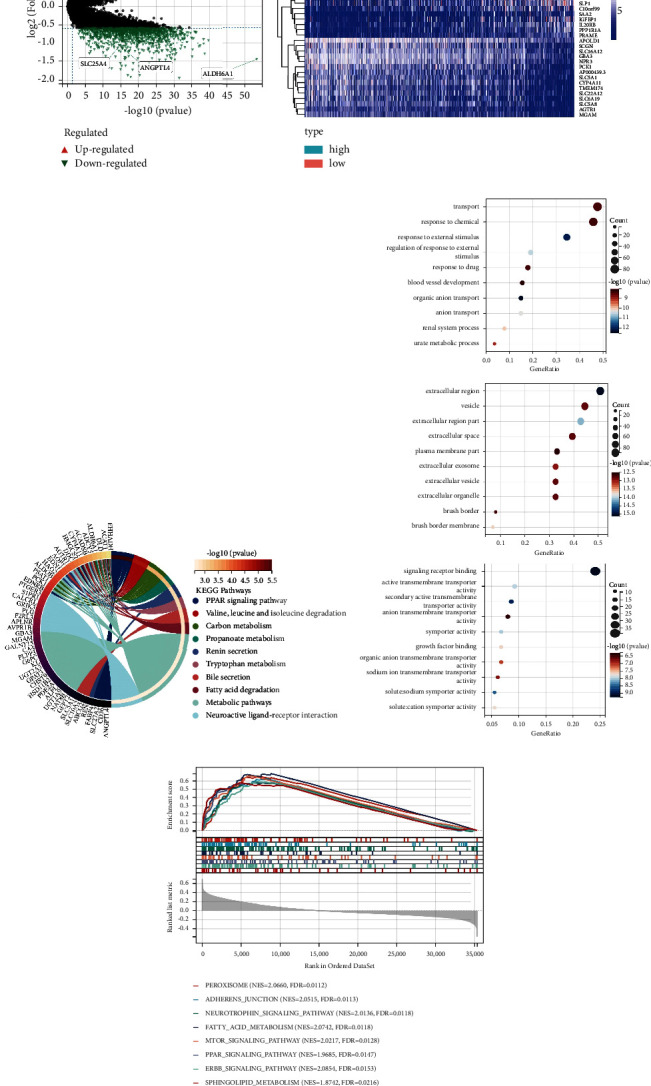
The exploration of the signaling pathways influenced by the hybrid metabolic activity. (a) The differential genes of high risk and low risk groups were displayed, and the differential genes (|Fold Chage| ≥ 1.5, *P* < 0.05) were marked in color. (b) The heatmap of the top 15 differential genes with high and low expressions. (c) The presentation of the results of KEGG analysis of the differential gene set. (d) The presentation of the results of the GO analysis of the differential gene set (cell component: top; molecular function: middle; and biological process: bottom). (e) The presentation of the results of GSEA analysis in the high-risk group.

To further clarify the effect of the hybrid metabolic activity on signal pathways and the corresponding functions of tumor cells in RCC, we conducted enrichment analysis based on the differential genes (Figures 4(a) and 4(b)) between the high- and low-risk groups and demonstrated the differential signaling pathways with the most prominent differences or that may have a certain research value.

KEGG analysis shows that lipid metabolism-related signaling pathways such as peroxisome proliferator-activated receptor (PPAR), fatty acid degradation, and amino acid metabolism signaling pathways such as leucine, tryptophan, and valine were significantly enriched (Figure 4(c)). In the GO analysis, the cell component analysis suggests that the hybrid metabolic activity affected the biological processes that mainly occurred in extracellular regions. Molecular function analysis enriched the cell's active transmembrane transport activity and signal receptor binding the related signaling pathways. Biological process analysis further confirmed that the transmembrane transport of tumor cells was significantly altered, and the response and regulation of cells to external stimuli such as drugs and chemicals were also significantly affected (Figure 4(d)).

In addition, to capture the effect of subtle coordination changes between genes on biological signaling pathways, we performed GSEA analysis based on patient subgroups developed by the hybrid metabolic activity-related prognostic model and the gene expression matrix and identified significant enrichment signaling pathways in high-risk patients (Figure 4(e)). Among them, the peroxisome signaling pathway involved in intracellular material transport and catabolism and the adhesion junction signaling pathway related to cell movement and intercellular connection were significantly enriched. Meanwhile, the mTOR and ERBB receptor tyrosine kinase signaling pathways related to RCC therapies were also significantly correlated with the confessor metabolic activity. The lipid metabolism signaling pathways mentioned in the previous analysis, such as fatty acid and sphingomyelin metabolism, were enriched again.

### 3.4. Effect of Hybrid Metabolic Activity on Tumor Immune Microenvironment in RCC

**Figure 5:**
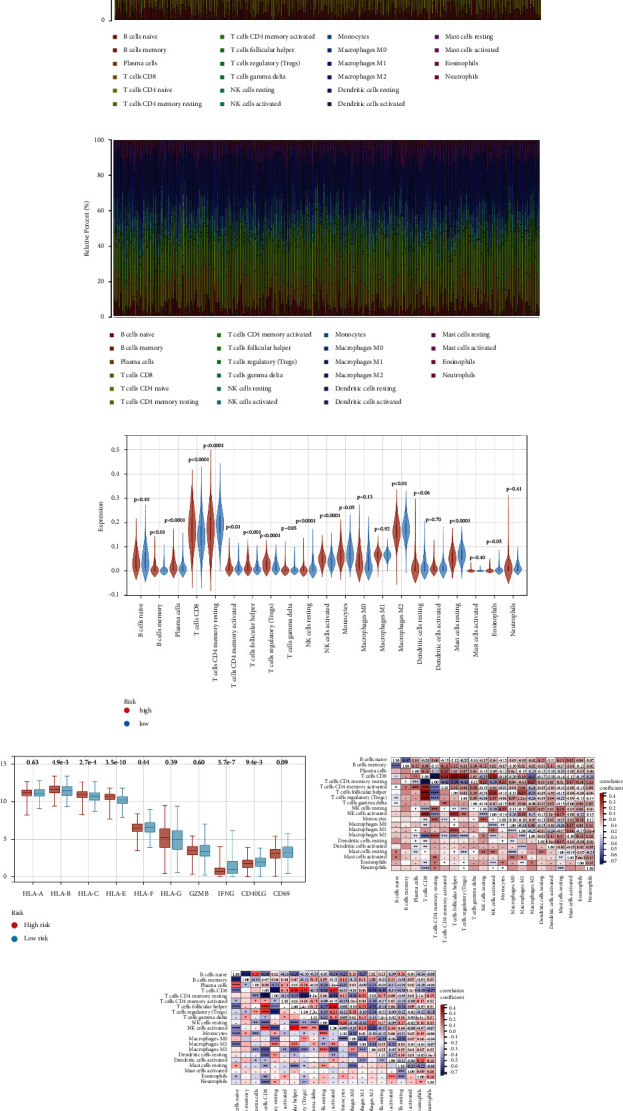
The effect of the hybrid metabolic activity on the immune cell infiltration in RCC. A&B: the proportion of various immune cells infiltrated in the tumor tissue in patients in the high-risk group (a) and the low-risk group (b). (c) The violin diagram of the results of analysis of differences in the proportion of infiltrated immune cells in tumor tissue between high risk and low risk groups. The comparison between the two groups was conducted based on the group *T* test. (d) Boxplot of gene expression levels in samples from high risk and low risk groups. The comparison between the two groups was conducted based on the group *T* test. E&F: results of correlation analysis of various immune cells infiltrated in tumor tissues of high risk (e) and low risk (f) groups (^*∗*^*P* < 0.05, ^*∗∗*^*P* < 0.01, ^*∗∗∗*^*P* < 0.001, ^*∗∗∗∗*^*P* < 0.0001).

The metabolic reprogramming of tumor cells often affects the differentiation, proliferation, and function of immune cells in the TME. Therefore, we delineated the immune cell infiltration in the RCC of patients in the high- (Figure 5(a)) and low-risk (Figure 5(b)) groups and investigated the differences in immune cell infiltration between the two groups using the paired *T* test. Combined with the bar diagram and the corresponding violin diagram (Figure 5(c)), we found that most T cell types, including CD8+T cells and regulatory T cells, greatly the tumor tissues of patients in the high-risk group except for memory CD4+T cells in the resting state. Patients in the high-risk group also have more active NK cells in their tumor tissue. In low-risk patients the overall macrophages infiltration is higher, especially in the M2 subtype. Considering the difference in the infiltration of CD8+T cells between high and low risk groups, we compared the expression of CD8+T cell function-related genes (GZMB, IFN-*γ*, CD40LG, CD69) and human MHC class I genes, including classic Ia genes (HLA-A, B, C) and nonclassic Ib genes (HLA-D, E, F), in high and low risk groups (Figure 5(d)). The results show that the expressions of HLA-B, C, and E are higher in the high-risk group, while the expressions of IFN-*γ* and CD40LG are higher in the low-risk group. To understand the association between various types of immune cells that infiltrated ccRCC, we also conducted a correlation analysis (Figures 5(e) and 5(f)). Interestingly, a significant positive association between CD8+T and Treg cells was found in both the high risk and low risk groups, and the association was stronger in the high-risk group. Memory CD4+T cells were also strongly associated with CD8+T cells in the high-risk group. The differences in the infiltration of these immune cells suggest that hybrid metabolic activity significantly alters the immune microenvironment of ccRCC.

### 3.5. Differences in Tumor Mutation Landscape in Patients with Different Hybrid Metabolic Activities and Possible Therapeutic Targets

**Figure 6:**
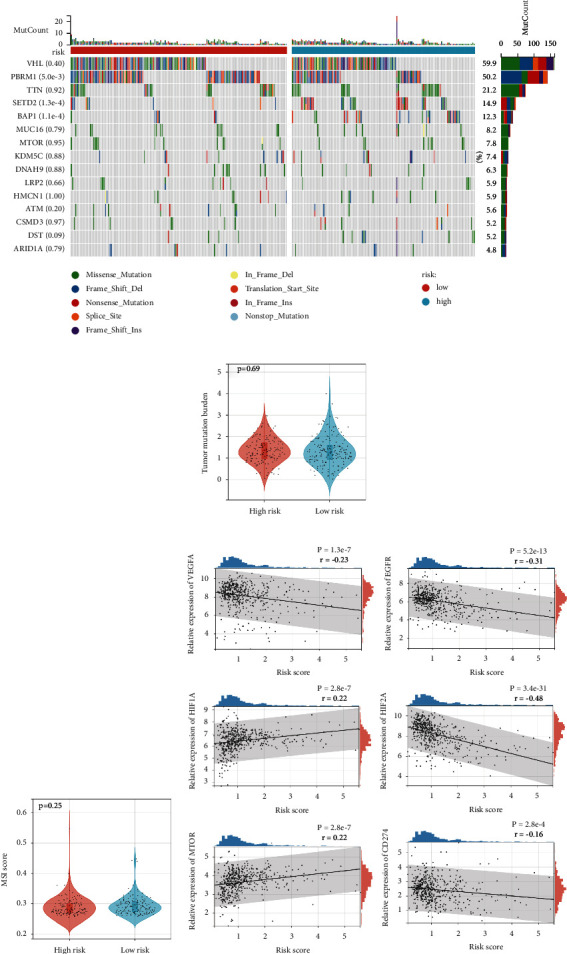
The association of the hybrid metabolic activity with the tumor mutations in RCC and exploration of possible therapeutic options. (a) The mutation landscape and difference analysis of genes with high mutation rate in the renal carcinoma in high and low risk groups. B&C: violin diagrams of differences in tumor mutation burden and microsatellite instability (MSI) between high and low risk groups. (D) The linear regression analysis of the risk score and expressions of target genes related to the treatment for RCC (VEGFA, EGFR, HIF1A, HIF2A, mTOR, and CD274).

We describe the mutation profiles of the top 15 genes with the most mutations in ccRCC in the high and low risk groups (Figure 6(a)). After comparison by the chi-square test, we found that the mutation incidence of STED2 and BAP1 was higher in the high-risk group, while the mutation incidence of PBRM1 was higher in the low-risk group. We then compared the overall tumor mutation burden (Figure 6(b)) and the MSI (Figure 6(c)) between the two groups and found no statistical differences.

Considering that targeted therapy and immunotherapy are the classical systemic therapies for ccRCC, they may also be potential therapeutic options for patients with high hybrid metabolic activity. We plotted the linear relationship between these target genes and the risk score, and the results show that the expressions of HIF1A and mTOR are significantly positively correlated with the risk score, while VEFFA, EGFR, HIF2A, and CD274 were negatively correlated with the risk score (Figure 6(d), Table S4). This result suggests that drugs targeting HIF1A and mTOR may be more suitable for these patients with a high hybrid metabolic activity.

### 3.6. Weakening of Metabolic Plasticity May Affect the Metastatic Ability of Renal Cell Carcinoma

**Figure 7:**
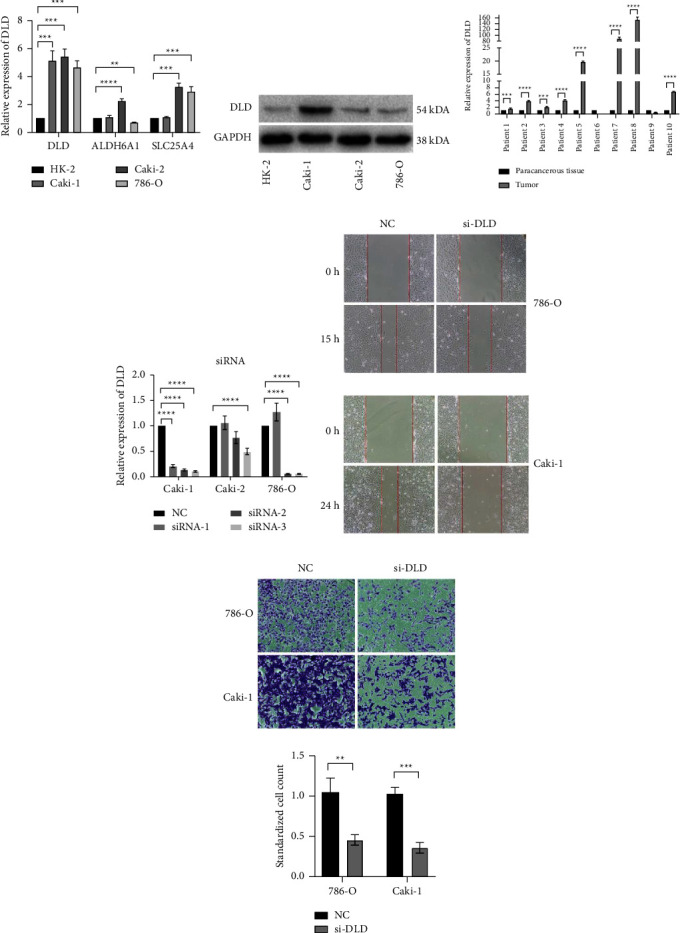
The validation of the expression of differentially expressed genes related to the hybrid metabolic activity and the effect of the DLD gene knockdown on the cell function in RCC. (a) The relative expression levels of DLD, ALDH6A1, and SLC25A4 genes in HK-2, Caki-1, Caki-2, and 786-O cell lines were detected by the qRT-PCR. (b) The expression levels of DLD gene encoded protein in HK-2, Caki-1, Caki-2, and 786-O cell lines were detected by the western blot. (c) The relative expression levels of DLD gene detected by the qRT-PCR in ten pairs of paired RCC tissues and adjacent tissues. (d) The expression level of DLD gene in 786-0 and Caki-1 cell lines was knocked down by siRNA, and the knockdown efficiency was verified by the qRT-PCR. (e) The scratch wound-healing assay revealed that DLD knockdown inhibited the migration of tumor cells of RCC. The images were taken at 20x. (f) The Transwell assay in 786-O and Caki-1 cell lines further demonstrated that DLD knockdown inhibited the migration of tumor cells. The images were taken at 20x. (^*∗*^*P* < 0.05, ^*∗∗*^*P* < 0.01, ^*∗∗∗*^*P* < 0.001, ^*∗∗∗∗*^*P* < 0.0001).

Previous analyses have shown that DLD, ALDH6A1, and SLC25A4 genes have a prominent impact on the survival and prognosis of patients with ccRCC in univariate analysis (Figure 3€). We used qRT-PCR to examine the transcriptional differences of these three genes in the normal renal cell line HK-2 and in RCC cell lines Caki-1, Caki-2, and 786-O (Figure 7(a)). The results show that the expression of the DLD gene in the three RCC cell lines was significantly higher than that in the normal renal tissue cell line and we further validated this finding at the protein level (Figure 7(b)). The expression trend of the DLD gene in most of the paired tissues of renal cell carcinoma patients is also consistent with the above results (Figure 7(c)).

Then, we explored the effect of the DLD gene expression on the function of RCC cells in-situ 786-O and metastasis Caki-1 by knocking down its expression (Figure 7(d)). Unfortunately, cck-8 assay (Figure S2A, S2B) and cell cloning (Figure S2C) experiments revealed that the DLD knockdown had no significant effect on tumor proliferation ability. In cell wound healing experiments, we found that the knockdown of the DLD gene expression significantly impaired the migration ability of the 786-O and Caki-1 cells (Figure 7(e)). In the subsequent Transwell experiment, our results confirm this phenotypic difference again (Figure 7(f)). This result may imply that the high hybrid metabolic activity in ccRCC may promote tumor metastasis.

## 4. Discussion

An important characteristic that differentiates cancer cells from normal cells is the reprogramming of the glucose metabolism, which allows them to dynamically switch between glycolysis and OXPHOS under hypoxic or aerobic conditions, allowing cancer cells to survive in different TMEs [19]. Under aerobic conditions, tumor cells usually preferentially metabolise glucose through the glycolysis pathway to obtain ATP efficiently, namely, the Warburg effect, and this phenomenon mainly occurs in highly proliferative tumor cells [20]. Recently, the up-regulation of peroxisome proliferator-related genes has been found to promote mitochondrial biosynthesis and OXPHOS and is associated with a more aggressive phenotype of tumors [21], which is also consistent with the results of our enrichment analysis and cell experiments. In addition, a single-cell sequencing analysis study also reported that primary tumors showed higher glycolysis metabolism, while micrometastases exhibited relatively higher OXHPOS metabolism [22]. Therefore, under the influence of reactive oxygen species (ROS), oncogene activation, and TME stimulation, cancer cells with a dual hybrid metabolic activity could continuously switch the preferred glucose metabolic pathway for the development of tumors from the initial growth to late metastasis [5, 23, 24]. However, studies on the hybrid metabolic activity are basically blank in RCC, and its related potential therapeutic targets and prognostic markers have not been revealed. Although drugs targeting the key molecules or genes of the metabolic pathway, such as glutaminase and HIF-2*α*, have been developed and applied in clinical trials for RCC, their objective response rate and safety are unsatisfactory [25, 26], highlighting the necessity of our study.

Among the genes included in the model and significantly related to survival, the expression of the DLD gene in the three types of RCC cell lines was higher than that in the normal cell line. The dihydrolipoamide dehydrogenase encoded by the DLD gene is a homologous flavin-dependent enzyme that catalyses NAD^+^-dependent dihydrolipoamide oxidation. It is involved in regulating apoptosis by producing ROS, and its enzyme activity is associated with tumor and apoptotic cell death [27]. We hypothesised that higher expression of the DLD gene in the high-risk group might indicate more ROS production. The oxidative stress in the TME may promote the formation of the hybrid metabolic activity in the high-risk group, suggesting that their tumors have a stronger ability to adapt to the TME. Cell experiments in this study suggest that the DLD gene is abnormally, highly expressed in RCC, and participates in regulating the migration ability of tumor cells. In conclusion, the DLD gene may promote the production of ROS to form a state of oxidative stress in the tumor and then stimulate tumor cells to obtain metabolic plasticity to enhance their invasion ability.

In the subsequent pathway enrichment analysis, we found some meaningful signaling pathways. Previous studies related to RCC have shown that the PPAR*α*, *γ* involved in the PPAR signaling pathway are involved in regulating the aggressiveness of tumor cells and may be used as prognostic markers of RCC [28, 29]. Moreover, a breast cancer study revealed that PPAR-gamma coactivator 1 alpha (PGC-1*α*) promotes cancer cell metastasis by mediating mitochondrial biosynthesis and OXPHOS (21). The increased OXPHOS activity of cancer cells induced by SETD2 deletion in RCC may also be associated with the PGC1*α* mediated metabolic network [30], which is consistent with the high SETD2 mutation rate found in the high-risk group. The mTOR signaling pathway, as a classic targeted molecule for the systemic therapy of RCC, has also been shown to promote and regulate the proliferation and invasion of cancer cells and is regulated by ECHS1, a key enzyme in fatty acid metabolism [31]. In addition, studies on breast cancer have shown that during the collective migration of tumor cells, the following cells guided by the lead cells will show a high level of intercellular adhesion, which also depends on the high expression of the adhesion junction signaling pathway [32].

Metabolic reprogramming can occur not only in tumor cells but also in immune cells, thus affecting the infiltration and function of immune cells [33]. Our study found more infiltration of antitumor immune cells and infiltration of immunosuppressive T cells, known as Tregs in the high-risk group. This seems to indicate that the RCC in the high-risk group is more immunogenic, presenting more tumor neoantigens to promote the activation of antitumor immune cells and chemotaxis to the TME. Studies on immune cells have shown that consistency with nonproliferative cells, OXPHOS is the dominant metabolic pathway in resting naive T cells. However, when T cells encounter tumor neoantigens, they change into a stronger glycolytic activity to enhance the proliferation and immune function [34], and during T cell chemotaxis, they must also constantly switch preferred metabolic pathways to face the constantly changing microenvironment. In addition, studies have shown that NK cells with a hybrid metabolic activity also have stronger antitumor activity [35]. Therefore, we consider that the TME of the RCC with a hybrid metabolic activity will infiltrate more antitumor immune cells. However, the presence of the Warburg effect causes tumor cells to consume large amounts of nutrients in the TME, thus placing metabolic limitations on T cells [36]. Glucose deprivation can prevent T cells in the TME from secreting cytokines to prevent them from further exerting the function of specific tumor killing [37]. Meanwhile, studies have shown that selective deletion of AMP-activated protein kinase, a key enzyme in the OXPHOS metabolic pathway, can inhibit the antitumor effect of CD8+T cells by inhibiting the secretion of IFN-*γ* and granzyme B [38]. In addition, CD4+Treg cells mainly rely on OXPHOS and lipid oxidation rather than glucose consumption to produce ATP, indicating that the TME of RCC with hybrid metabolic activity is more suitable for its function of limiting antitumor immunity [39]. These studies, together with the comparison of the expression of genes related to MHC-I molecules, granzyme B, etc., suggested that although the RCC in the high-risk group may infiltrate more immune cells, the antitumor immune function of these cells may be significantly limited. However, the patients in the high-risk group may be better candidates for immunotherapy that promotes the activation of CD8+T cells and improves their cytotoxicity, as well as inhibits the expansion of Treg [40, 41].

In the mutation landscape difference analysis, in addition to SETD2 mentioned above, BAP1 also had a significantly higher mutation rate in the high-risk group. RCC with BAP1 mutation has significant morphological overlap with Xp11 translocation RCC and is also associated with a higher tumor stage. Renal vein invasion is common in this type of RCC and the rate of metastasis is up to 50% [42]. PBMR1, which exhibits a significantly higher mutation rate in the low-risk group, was found to be associated with poorer survival in patients receiving immunotherapy [6]. The deletion of PBMR1 induces the formation of a nonimmunogenic tumor microenvironment in RCC and reduces the presentation of tumor neoantigens, thus inducing resistance to immune checkpoint blocking therapy [43], which is consistent with our results in immune cell infiltration analysis. In addition, in the analysis that searched for possible therapeutic targets, we found that HIF-1*α* and mTOR had a significant positive correlation with the risk score. HIF-1*α*, as an important subunit of HIF-1, is closely involved in the adaptive response of tumor cells to a hypoxic environment [44]. The high expression of mTOR can also upregulate the expression of HIF-1*α* and stimulates the function of its transactivation domain [45]. These two genes may be important targets to block the formation of metabolic plasticity in tumors of the high-risk group.

In this study, we constructed a prognostic model with great predictive efficacy. Furthermore, we explored the effect of the hybrid metabolic activity on the tumor cells in RCC from the aspects of the signaling pathway, immunity, tumor mutation, and cell function. We found that the hybrid metabolic activity may be involved in tumor migration in several aspects and explored possible therapeutic options and prognostic indicators for patients with this type of RCC. Although the underlying mechanism is not fully revealed, this study may be helpful for further studies on the metabolic aspects of RCC.

## Figures and Tables

**Table 1:** The clinical characteristics, pathological characteristics, and follow-up outcomes of the patients included in the training set. AJCC: American Joint Committee on Cancer.

Characteristics	Group	Patients (*n*, %)
Age	≥65	193 (36.76)
	<65	332 (63.24)

Gender	Male	343 (65.33)
	Female	182 (34.67)

Grade	1-2	239 (45.52)
	3-4	278 (52.95)
	Unknown	8 (1.52)

AJCC stage	I-II	317 (60.38)
	III-IV	205 (39.05)
	Unknown	3 (0.57)

T satge	T1-T2	335 (63.81)
	T3-T4	190 (36.19)

N stage	N0	237 (45.14)
	N1	16 (3.05)
	Unknown	272 (51.81)

Metastasis	M0	417 (79.43)
	M1	78 (14.86)
	Unknown	30 (5.71)

Survival status	Alive	361 (68.76)
	Dead	164 (31.24)

## Data Availability

All data come from publicly available databases. We annotated the data sources in the article. For further information, please email the corresponding author.

## Supplementary Materials

Supplementary Materials Table S1: Sequence of primers for the qRT-PCR experiment. Table S2: Statistical parameters of the hybrid metabolic activity-related prognostic model. Table S3: Clinical data of patients in the validation set. Table S4: Statistical parameters of the correlation analysis in Figure 6(d). Figure.S1: The batch effect removal of the validation set.Figure.S2: The effect of the DLD gene on the proliferation ability of renal carcinoma cells.
