# Supplementary material for: Hybrid Metabolic Activity-Related Prognostic Model and Its Effect on Tumor in Renal Cell Carcinoma
Source: J Healthc Eng. 2022 Dec 21;2022:1147545. doi: 10.1155/2022/1147545 (PMC9797315; doi:10.1155/2022/1147545)
Supplement: Supplementary Materials — Table S1: Sequence of primers for the qRT-PCR experiment. Table S2: Statistical parameters of the hybrid metabolic activity-related prognostic model. Table S3: Clinical data of patients in the validation set. Table S4: Statistical parameters of the correlation analysis in Figure 6(d). Figure.S1: The batch effect removal of the validation set.Figure.S2: The effect of the DLD gene on the proliferation ability of renal carcinoma cells. [file 1147545.f1.docx]

**Supplementary materials**

**Supplementary tables:**

| ID | Primer sequence | |
| --- | --- | --- |
| DLD | Forward | CACTGCTACGAAAGCTGATGG |
|  | Reverse | TAACTTCTGAACCCGTGGCTA |
| ALDH6A1 | Forward | GGCAGACACTTCAGTATTAAGCC |
|  | Reverse | AGAGGCAGACGGTAGGAATAAA |
| SLC25A4 | Forward | ATGGAATGGGAACGTCTGTTG |
|  | Reverse | CCTCTCGGACGCACAGGATA |
| Actin | Forward | GACGGGGTCACCCACACTGTGCCCATCTA |
|  | Reverse | CTAGAAGCATTTGCGGTGGACGATGGAGGG |

Table.S1 Sequence of primers used to amplify the target gene of cDNA in qRT-PCR experiment.

| **Gene** | **Coefficient** | **Standard error(coefficient)** | **P value** |
| --- | --- | --- | --- |
| ALDH6A1 | -0.2968 | 0.7432 | 0.0036 |
| ANGPTL4 | -0.1879 | 0.8287 | 0.0107 |
| DLD | 0.1895 | 1.2086 | 0.0446 |
| NNT | -0.2116 | 0.8093 | 0.1029 |
| PDHA1 | 0.3843 | 1.4687 | 0.1065 |
| PHKA2 | 0.2912 | 1.3380 | 0.0138 |
| SLC25A4 | -0.4706 | 0.6246 | 0.0023 |
| SUCLG1 | 0.4357 | 1.5461 | 0.0390 |

Table.S2 The coefficients, standard errors of these coefficients and P values of each factor in the hybrid metabolic activity-related prognostic model.

| **Characteristics** | **Group** | **Patients(n,%)** | | |
| --- | --- | --- | --- | --- |
| **Data set** |  | **RECA-EU** | **CCSK-US** | **WT-US** |
| **Age** | ≥65 | 54(39.71%) | NA | NA |
|  | ＜65 | 82(60.29%) |  |  |
| **Gender** | Male | 80(58.82%) | 10(76.92%) | 2(25.00%) |
|  | Female | 56(41.18%) | 56(23.08%) | 6(75.00%) |
| **Grade** | 1-2 | 19(13.97%) | 4(30.77%) | 3(37.50%) |
|  | 3-4 | 27(19.85%) | 9(69.23%) | 5(62.50%) |
|  | Unknown | 90(66.18%) | 0(0.00%) | 0(0.00%) |
| **T satge** | T1-T2 | 98(72.06%) | NA | NA |
|  | T3-T4 | 38(27.94%) |  |  |
| **N stage** | N0 | 116(85.29%) | NA | NA |
|  | N1 | 3(2.21%) |  |  |
|  | Unknown | 17(12.50%) |  |  |
| **Metastasis** | M0 | 120(88.24%) | NA | NA |
|  | M1 | 14(10.29%) |  |  |
|  | Unknown | 2(1.47%) |  |  |
| **Survival status** | Alive | 89(64.03%) | 9(69.23%) | 6(75.00%) |
|  | Dead | 47(35.56%) | 4(30.77%) | 2(25.00%) |
| **Total** |  | 136 | 13 | 8 |

Table.S3 The clinical characteristics, pathological characteristics and follow-up outcomes of the patients included in the validation set.

| X | Y | Equation | Slope | | 95% Confidence Interval | R | P value |
| --- | --- | --- | --- | --- | --- | --- | --- |
| Risk score | VEGFA | Y = -0.3629*X + 8.570 | -0.3629 | -0.4960 to -0.2299 | | -0.23 | 1.3E-07 |
|  | EGFR | Y = -0.4131*X + 6.555 | -0.4131 | | -0.5227 to -0.3036 | -0.31 | 5.2E-13 |
|  | HIF1A | Y = 0.2277*X + 6.180 | 0.2277 | | 0.1418 to 0.3136 | 0.22 | 2.8E-07 |
|  | HIF2A | Y = -0.6790*X + 9.004 | -0.679 | | -0.7864 to -0.5717 | -0.48 | 3.4E-31 |
|  | MTOR | Y = 0.1591*X + 3.480 | 0.1591 | | 0.0991 to 0.2191 | 0.22 | 2.8E-07 |
|  | CD274 | Y = -0.1526*X + 2.617 | -0.1526 | | -0.2346 to -0.07061 | -0.16 | 0.00028 |

Table.S4 The equation, slope, confidence interval and other parameters of correlation analysis between genes and the risk score in Figure 6D.

**Supplementary figures:**


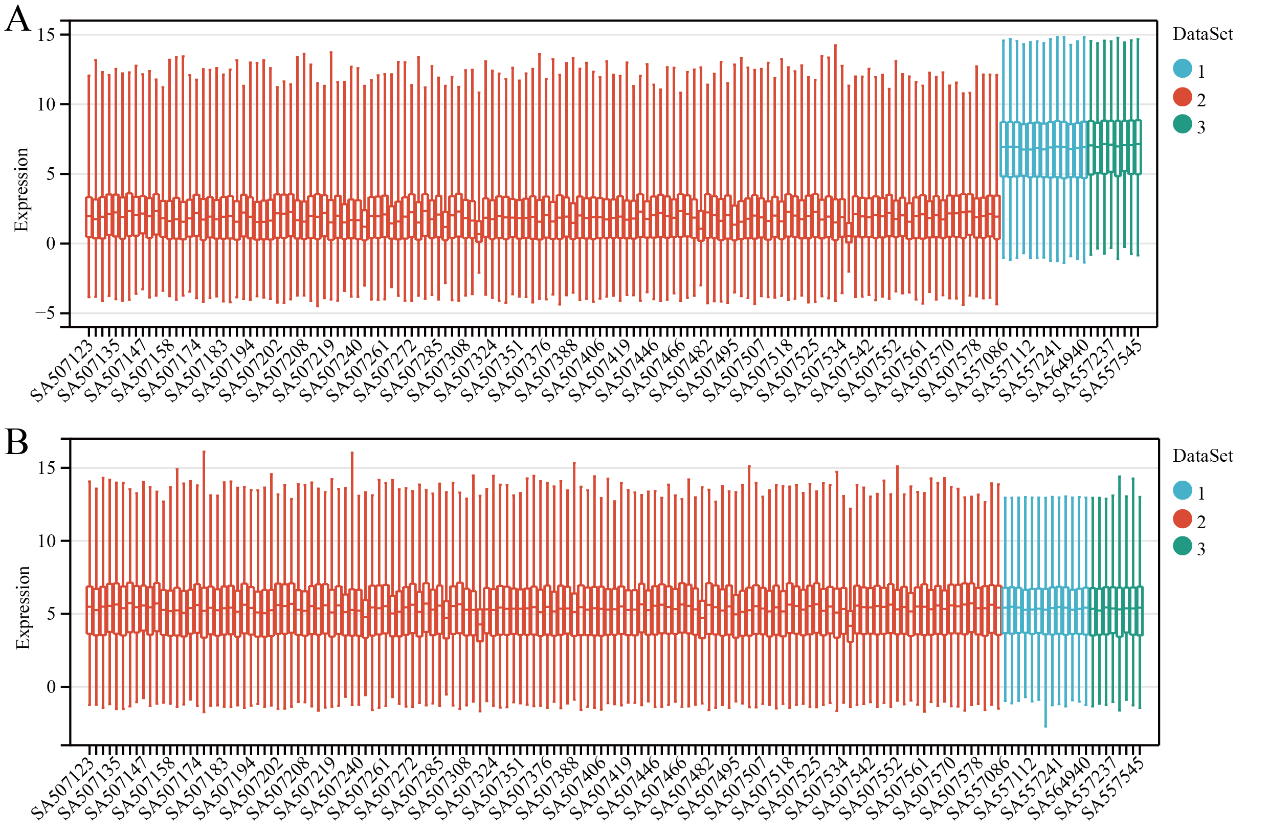


Figure.S1 Batch effect removal of the validation set. A＆B: Boxplot of expression distribution of samples from three data sets before (A) and after (B) removing the batch effect.


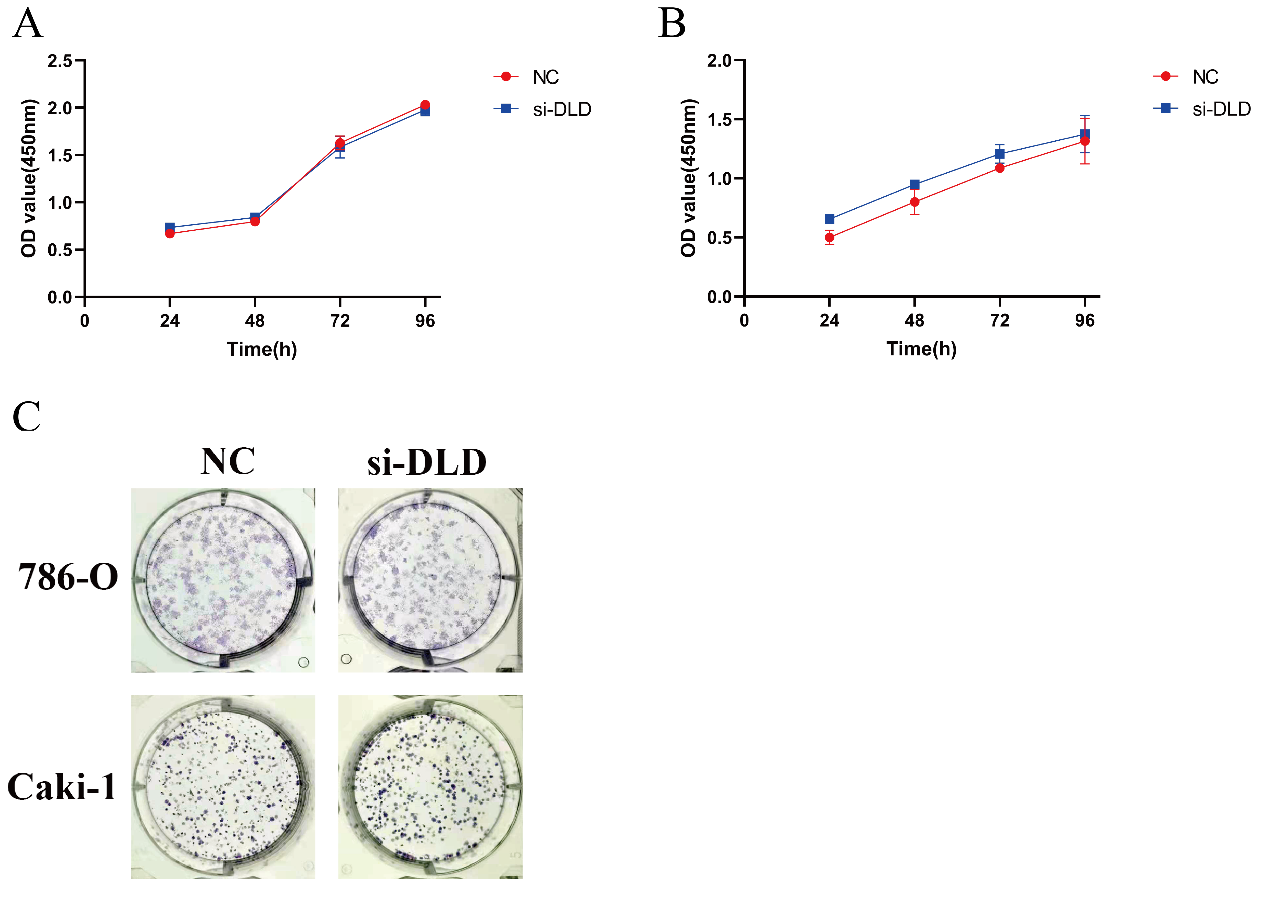


Figure.S2 Exploration of the effect of DLD gene knockdown on the proliferation ability of renal carcinoma cells. A&B: CCk8 assay in 786-O (A) and Caki-1 (B) cell lines showed that DLD knockdown had no significant inhibitory effect on the proliferation of tumor cells. C: Cell cloning experiments in 786-O and Caki-1 cell lines also showed that DLD knockdown had no significant effect on the clonogenic ability of tumor cells. (*P < 0.05, **P < 0.01, ***P < 0.001, ****P< 0.0001)
